# Depression, glycemic control and type 2 diabetes

**DOI:** 10.1186/1758-5996-3-26

**Published:** 2011-10-07

**Authors:** Marcelo Papelbaum, Rodrigo O Moreira, Walmir Coutinho, Rosane Kupfer, Leão Zagury, Silvia Freitas, José C Appolinário

**Affiliations:** 1 Obesity and Eating Disorders Group, Psychiatry Institute of the Federal University of Rio de Janeiro, Rio de Janeiro, 22290-140, Brazil; 2 Department of Diabetes, State Institute of Diabetes and Endocrinology of Rio de Janeiro, Rio de Janeiro, 20211-340, Brazil

## Abstract

**Background:**

Comorbid depression in diabetes has been suggested as one of the possible causes of an inadequate glycemic control. The purpose of this study was to investigate the association between major depression and the glycemic control of type 2 diabetes mellitus (T2DM).

**Methods:**

Seventy T2DM patients were evaluated. They underwent a psychiatric examination using the following instruments: Structured Clinical Interview for DSM-IV and Beck Depression Inventory. The diabetes status was assessed in the short-term (glycemia, glycated hemoglobin) clinical control.

**Results:**

The presence of current depression was observed in 18.6% (13/70). In addition, type 2 diabetes patients who displayed depression evidenced higher levels of glycated hemoglobin (8.6 ± 2.0 vs. 7.5 ± 1.8; p = 0.05) when compared to those who did not exhibit a mood disorder.

**Conclusions:**

In our sample, the presence of depression seems to impact on the short-term control of T2DM. The authors discuss the clinical utility of these findings in the usual treatment of diabetes.

## Background

According to the American Diabetes Association (ADA) guidelines, patients with diabetes should maintain low levels of glycated hemoglobin (A1C), blood pressure and low-density lipoprotein [1]. However, only 10% of the patients meet all those three goals simultaneously [2]. As a consequence, the control of the disease is inadequate, leading to an increase of the morbidity and mortality [3].

Several factors may account for an unsatisfactory metabolic control of DM. Among them it is important to emphasize the relevance of the occurrence of a psychiatric comorbidity. The presence of depression in a patient with diabetes has been suggested as one of the possible causes of an inadequate metabolic control, especially for those patients who cannot achieve an adequate glycemic control despite intensive medical recommendations.

Several studies observed higher rates of depression in patients with diabetes and tried to demonstrate the association between the presence of depressive symptoms and an increased prevalence of clinical complications of the DM. Anderson et al. conducted a meta-analysis of 42 studies investigating the association between depression and diabetes [4]. The authors found that the presence of diabetes doubles the odds of having depression. This risk remained even after controlling for both types of diabetes or different diagnostic methods for depression across studies. O'Connor et al. followed a retrospective cohort of patients with diabetes to investigate the prevalence of depression [5]. The authors observed an increase prevalence of depression in patients with diabetes compared to a non-diabetic sex- and age-matched control group.

Furthermore, several authors found an association between depression and a poor metabolic control of T2DM. In a meta-analytic review of the literature, Lustman et al. investigated the association between depression and diabetes glycemic control [6]. The authors observed that depression was significantly associated with hyperglycemia in patients with type 1 and type 2 diabetes (z = 5.4; p < 0.0001). In the same line, de Groot et al., conducted a meta-analysis examining the association between depression and diabetes outcomes [7]. Twenty-seven studies were included and the authors found a significant association between depression and clinical complications of diabetes (p < 0.00001; z = 5.94).

The purpose of this study was to investigate the association between depression and the clinical control of T2DM with respect to short-term (glycemia and A1C levels) complications in a Brazilian sample of patients with diabetes.

## Methods

Seventy type 2 diabetic outpatients, aged 30 to 65 years, were assessed consecutively at the State Institute of Diabetes and Endocrinology of Rio de Janeiro. T2DM was diagnosed according to ADA criteria [8]. All patients underwent clinical and psychiatric evaluation. Exclusion criteria included the presence of type 1 diabetes, gestational diabetes or secondary diabetes due to another disease, the use of medications likely to affect food intake (appetite suppressants and other anti-obesity drugs) and the incapacity to self-complete the questionnaires. The Ethics Committee of the Institution approved the protocol and written informed consent was obtained after the procedures involved in the study were fully explained.

### Clinical Examination

Anthropometric examinations were made and laboratory tests were obtained to assess the metabolic control of diabetes. For the short-term evaluation, fasting glycemia and A1C levels were assessed. Glycemia was measured using a colorimetric enzymatic method, and A1C levels were determined using high-performance liquid chromatography (HPLC; Variant turbo, Bio-Rad, National Glycohemoglobin Standardization Program).

### Psychiatric instruments

All subjects were interviewed by a psychiatrist (MP) trained in the use of the Structured Clinical Interview for DSM-IV, patient edition (SCID-P) [9]. This instrument was used for the current and past diagnosis of depression. The Beck Depression Inventory (BDI) was also applied to provide a measure of severity of general psychopathology [10]. Two studies evidenced that the BDI was an effective screening test for major depression in diabetic patients. Lustman et al. observed that both cognitive and somatic items exhibited satisfactory psychometric properties [11]. In another study, Hermanns et al. screened 376 diabetic patients for clinical depression [12]. The authors observed that the BDI presented the best sensitivity (87%), compared to Center of Epidemiological Studies-Depression Scale (79%) and the Problem Areas in Diabetes questionnaire (81%), which assesses diabetes-specific distress. In addition, the BDI has been translated and validated into Portuguese, presenting good reliability [13].

### Procedures

Patients who met the inclusion criteria were consecutively evaluated. They were asked to complete the BDI, and the SCID-P examination was performed. Laboratory tests were subsequently performed.

### Statistical Analysis

Continuous variables were expressed as means and standard deviation, and categorical data were expressed as absolute and relative frequencies. Patients with depression were compared to those with no general psychopathology in terms of clinical and psychiatric characteristics. The Mann-Whitney test was used to compare means of continuous variables, and the Chi-square test was used to analyze categorical variables. Correlations of BDI scores with glycemia and A1C levels were made using Spearman's test. A significance level of 5% was adopted.

## Results

In our sample, current depression was evidenced in 18.6% (13/70) of the patients. Also, almost 36% of the patients (25/70) of the patients had a past history of mood disorder, using the SCID-P (Table 1). Furthermore, patients with current depression exhibited a greater severity of depressive symptoms, using the BDI, when compared to those who did not present depression at evaluation (22.9 ± 6.5 vs. 12.7 ± 9.0; p < 0.001). Also, none of the depressed patients was receiving specific psychiatric or psychological treatment. Table 1 displays several clinical characteristics of the studied population. It is important to note that, approximately half of the patients were using insulin at the time of examination.

**Table 1:** Clinical variables of type 2 diabetes mellitus patients

	N (%; median ± sd)
BMI (kg/m^2^)	30,56 ± 5.20
Normal	18.6
Overweight	31.4
Obese	50
Duration of diabetes (years)	13.36 ± 7.55
Insulin use	51.4
Glucose (mg/dl)	164.7 ± 75.9
Glycated hemoglobin (%)	7.72 ± 1.91
BDI	14.6 ± 9.5

BMI - Body Mass Index; BDI - Beck Depression Inventory

Patients who displayed depression were younger than those who did not exhibit a mood disorder (49.5 ± 6.9 vs. 53.7 ± 6.6; p = 0.04). In the same way, type 2 diabetic patients who evidenced current depression had higher rates of insulin use than those without depression (27.8% vs. 8.8%; p = 0.04).

**Figure 1:**
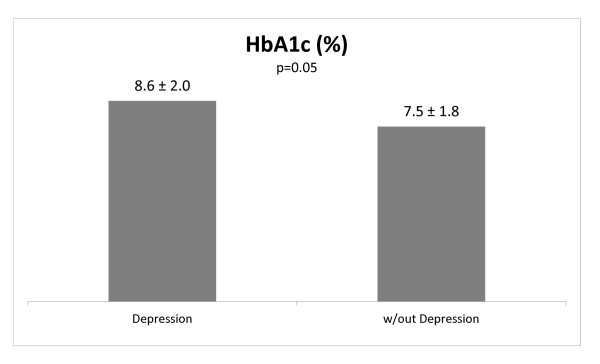
**Mean glycated hemoglobin (HbA1c) levels in type 2 diabetes patients with and without current depression**.

Patients with T2DM who had depression had a poorer glycemic control compared to patients without mood disorder (Figure 1), even after controlling for age and insulin use. Conversely, patients who exhibited lifetime depression, but were not depressed at the time of evaluation, did not display higher A1C levels compared to those patients without any past history of mood disorder (7.9 ± 2.0 vs. 7.6 ± 1.8; p = 0.57).

## Discussion

In our study, almost 20% of patients with T2DM exhibited current depression. Those diabetic patients with comorbid depression had significantly poor glycemic control compared to those without any mood disorder.

The prevalence of depression found in our study was similar to that observed in other investigations [14,15]. However, some studies had showed that the elevated rates of depression observed in diabetes were also associated to others clinical variables than the presence of diabetes. Pouwer et al. conducted a community-based cross-sectional study to evaluate the prevalence of depression in 216 subjects with diabetes and 1184 subjects without any chronic disease [16]. The authors found an increased prevalence of depression only in diabetic subjects who displayed a comorbid clinical disease. In another study, Kruse et al. compared the prevalence of mental disorders between 141 diabetic patients and 4,028 subjects without diabetes in community sample [17]. The authors observed that people with diabetes were not more likely to meet the DSM-IV criteria for depression than subjects without diabetes when the findings were controlled for age, sex, marital status and socioeconomic status (OR = 1.41, 95% CI [0.78-2.55]). Finally, in a recent study, O'Connor et al. evaluated the association between depression and the number of primary care visits in two large samples of diabetic patients and a non-diabetic control group [5]. The authors found a negative correlation between the risk for depression and the number of primary care visits the patient attended.

It seems important to note the absence of specific interventions to ameliorate depression in our sample of T2DM patients. Indeed, the subdiagnosing and undertreatment of depression in patients with comorbid physical illness has been reported in several studies, even after the implementation of clinical-practice guideline to improve recognition of depression in primary care [18,19]. In a recent review, beliefs from physicians that depression was a normal response to life events, the clinician struggle to differentiate distress from depression and the subnotification of depressive symptoms from the patients in order to avoid stigmatization, were some of the barriers identified in the adequate management of depression [20].

Besides the elevated prevalence of depression in diabetes, several authors observed an impact of depression on glycemic control of diabetes. Skaff et al., evaluating 206 type 2 diabetic patients, showed that a daily negative mood correlated positively with the fasting glucose level of the next morning in men with T2DM (r = 0.17; p < 0.05) [21]. In addition, Eren et al. examined the impact of depression on clinical control of 104 T2DM patients [22]. The authors observed that the number of depressive episodes correlated positively with A1C levels. A longitudinal study investigated the correlation between depression and A1C levels in T2DM patients with and without depression [23]. The authors observed that type 2 diabetic patients with depression exhibited higher A1C levels compared to patients without depression in all time points evaluated (mean difference of 0.13; 95% CI [0.03-0.22]; p = 0.008). In the same line with previous investigations, our study found that patients with diabetes who displayed depression had higher A1C levels compared to those without depression. However, patients with lifetime depression without depressive symptoms at evaluation did not exhibited higher A1C levels compared to those with clinical depression at examination. In a similar study, de Groot et al., evaluating 39 T2DM patients, also observed that type 2 diabetes patients with a lifetime history of major depression did not have significantly worse control than those with no history of psychiatric illness [24].

Although the use of insulin was not associated with glycemic control in our study, we observed that patients who were using insulin had elevated rates of depression compared to those who were using only oral hypoglicemiants. Aikens et al. investigated the influence of treatment regimen on the association between depression and A1C levels [25]. The authors, evaluating 258 T2DM patients, observed that depression was associated with A1C levels in patients using insulin (beta = 0.35; p < 0.001) but not in patients using oral agents alone (beta = -0.08; p = NS).

Some limitations of this study must be discussed. The sample size was relatively small. In addition, inclusion of others clinical variables such, number of clinical appointments and measures of medical and nutritional adherence group could have helped in better understanding the role of depression in our results. Also, the absence of control for antidepressant use could have biased the severity of depression in the sample. However, our findings add one more piece of information regarding the impact of depression on the metabolic control of diabetes. Furthermore, this is the first observational study on the association between depression and glycemic control of T2DM in a Brazilian clinical sample.

## Conclusions

Our study is in line with previous investigations that showed an inadequate glycemic control in the co-occurrence of depression and type 2 diabetes. Of note, the presence of psychiatric comorbidity in diabetic patients should always be investigated and a carefully monitoring of the metabolic control might be important of those individuals who exhibit clinical depression. Nevertheless, it would be advantageous to have other longitudinal studies to better understand the nature of those associations.

## List of Abbreviations

A1C: Glycated hemoglobin; ADA: American Diabetes Association; BDI: Beck Depression Inventory; T2DM: Type 2 diabetes mellitus; SCID-P: Structured Clinical Interview for DSM-IV, patient edition;

## Competing interests

No potential conflict of interest relevant to this article was reported.

This study was not funded, and as such there are no funding sources.

## Authors' contributions

MP, LZ and JCA participated in the design of the study and ROM and MP performed the statistical analysis. RK participated in its design and coordination and WC helped to draft the manuscript. All authors read and approved the final manuscript.
